# Evaluation of in vitro biological potential of plant species *Sebastiania corniculata *(Euphorbiaceae)

**DOI:** 10.1186/1753-6561-8-S4-P40

**Published:** 2014-10-01

**Authors:** Isabelle Souza de Mélo Silva, Raíssa Fernanda Evangelista Pires dos Santos, Andriele Mendonça Barbosa, Klebson Silva Santos, Mariene Ribeiro Amorim, Flavia Manuella Ribeiro de Mendonça, Ingridy Viana Lucena, Thiane de Vasconcellos Costa Melo, Genilson Sarmento Lins Júnior, Patricia de Albuquerque Sarmento, Francine Ferreira Padilha, Maria Lysete de Assis Bastos

**Affiliations:** 1 Instituto de Tecnologia e Pesquisa, Universidade Tiradentes - Av. Murilo Dantas, 300, Farolândia - Aracaju, Sergipe, CEP 49032-490, Brazil; 2 Laboratório de Pesquisa em Tratamento de Feridas, Universidade Federal de Alagoas, Campus A. C. Simões - Av. Lourival Melo Mota, s/n, Cidade Universitária - Maceió, Alagoas, CEP 57072-900, Brazil

## Background

Medicinal plants with antimicrobial properties built into the problem of multidrug deserve investment in research, by guiding the discovery of herbal medicine effective against emerging pathogens and related bacterial and fungal infection. Brazil has a great biodiversity of plants, which are popularly used for medicinal form. Vegetables have been widely used in health care due to its medicinal properties, such as antibacterial, antifungal and immunomodulatory activities [1]. Species of the family Euphorbiaceae are popularly used against diseases of viral, antimicrobial, anti-inflammatory, antiulcer, antihypertensive, muscle relaxant [2], including *Sebastiania macrocarpa, Sebastiania hispida, Sebastiania commersoniana*. However, works that related biological potential of *Sebastiania corniculata *species are scarce. Through this, the objective of this study was to evaluate the antimicrobial activity and toxicity of *Sebastiania corniculata*, guanxuma-de-chifre, popularly used as antidiarrheal, antibacterial and elimination of kidney stones [3].

## Methods

Experimental in vitro study, was conducted at the Laboratory of Wound Care at Federal University of Alagoas. Were evaluated two fractions of the species *Sebastiania corniculata *extract, X_A _and X_E_. 14 microorganisms were used, standardized between bacteria and fungi which were distributed by American Type Cell Collection. Antimicrobial activity was determined by microbial sensitivity tests, the method of disk diffusion (DD) and the method of broth microdilution for determination of minimum inhibitory concentration (MIC). All extracts were tested against *Artemia salina *Leach. For the study of cell viability, only the XA extract was tested.

## Results and conclusions

The extracts showed moderately active for *Staphylococcus aureus *and *Pseudomonas aeruginosa *in the DD test (percentage inhibition >28.6 to <51.38), confirmed by the results of CIM. The extract X_A _is the fraction of the best antimicrobial potention, inhibiting the growth of *P. aeruginosa *lineage, concentration of 1000 to 125 μg mL^- 1 ^by the result of the MIC. These results corroborate previous studies since species of the family *Euphorbiacea *[3], *Heterocalyx croton *and *Euphorbia hirta *[4], *Palidullus croton *and *Croton ericoides *, that also showed antimicrobial activities against these organisms [4]. Was identified the absence of toxicity in all samples, since the percentage of mortality was ≤30 %. This finding dismissed the realization of the quantitative assay. All extracts showed inactive against fungi tested, because it inhibited their growth. The low activity and/or the absence of antifungal activity may be due to the plant extracts tested did not damaged the membrane permeability to allow fungal cell. The evaluation of cytotoxicity by MTT, X_A _extract showed significant cytotoxicity (p < 0.0001). These results cooperates with MTT describes a study significantly increased compared to the antitumour agent 5-fluorouracil [5]. *In vitro *studies are the basis for further research of technological advancement involving the use of *S. corniculata *for therapeutic purposes, including as antimicrobial.
